# Systematic review of dengue vaccine efficacy

**DOI:** 10.1186/s12879-019-4369-5

**Published:** 2019-08-28

**Authors:** Lucia Teresa Côrtes da Silveira, Bernardo Tura, Marisa Santos

**Affiliations:** 1 Fire Department/Rio de Janeiro State and Professor of medicine at Unigranrio, NATS - Rua das Laranjeiras 374/ 5° andar CEP 22240-004, Rio de Janeiro, RJ Brazil; 2 Biostatistics and Modelling/ Instituto Nacional de Cardiologia, Rio de Janeiro, RJ Brazil; 3 PHD Epidemiology, Instituto Nacional de Cardiologia, Rio de Janeiro, RJ Brazil

**Keywords:** Dengue, Dengue vaccines, Dengue virus, Systematic review, Health technology assessment

## Abstract

**Background:**

Dengue is an arbovirus that has rapidly spread worldwide, and the incidence of dengue has greatly increased in recent decades. The actual numbers of dengue cases are underreported, and many cases are not classified correctly. Recent estimates indicate that 390 million dengue infections occur per year (95% CI, 284–528 million), of which 96 million (67–136 million) are symptomatic infections of any severity. One of the goals of the World Health Organization is to reduce dengue mortality by 50% by the year 2020. The use of a vaccine can be an important strategy to achieve this goal. Vaccines for dengue are in various stages of development; in Brazil, only one commercial formulation is available (CYD-TDV), which was developed by Sanofi Pasteur.

**Methods:**

To evaluate the efficacy of Dengue vaccine, a systematic review with a meta-analysis was conducted using randomized controlled clinical trials published between 2000 and 2017 that were identified in the MEDLINE databases via PubMed, LILACS, Cochrane Library, and EMBASE. The selection was performed by two reviewers independently, with disagreements resolved by a third reviewer.

**Results:**

Seven clinical trials were included, with a total of 36,371 participants (66,511 person-years) between the ages of 2 and 45 years. The meta-analysis using the random-effects model estimated the efficacy of the vaccine at 44%, with a range from 25 to 59% and high heterogeneity (I^2^ = 80.1%). The serotype-stratified meta-analysis was homogeneous, except for serotype 2, with the heterogeneity of 64.5%. Most of the vaccinated individuals had previous immunity for at least one serotype, which generated safety concerns in individuals without previous immunity.

**Conclusions:**

Compared with other commercially available vaccines, the dengue vaccine showed poor efficacy.

**Electronic supplementary material:**

The online version of this article (10.1186/s12879-019-4369-5) contains supplementary material, which is available to authorized users.

## Background

Dengue is a viral disease caused by one of four single-stranded RNA dengue viruses, serotypes dengue 1, dengue 2, dengue 3 and dengue 4 (serotypes DENV-1, DENV-2, DENV-3, and DENV-4). The virus belongs to the genus *Flavivirus*, family *Flaviviridae* [1] and is transmitted to humans by the bite of infected *Aedes* mosquitoes (mainly *Aedes aegypti*) [2].

Dengue is a disease of great importance for public health. A recent estimate indicated that globally, 390 million dengue infections occurred per year, of which 96 million clinically manifested infections [3]. Moreover, one of the goals of the World Health Organization is to reduce dengue mortality by 50% by the year 2020 [4]. In 2013, Dengue was estimated to have caused medical expenses in Brazil totalling US$1,227,551,975 according to a study sponsored by Sanofi Pasteur [5].

There is no specific treatment for dengue. The prevention of dengue infection is theoretically the best strategy and is currently performed mainly through vector control, which is a complex and inefficient action that is multifactorial and multisectoral. According to the WHO, ideally, a dengue vaccine should protect against all four serotypes, be given as a single dose, have long-term immunity and have no serious adverse effects [6].

The vaccine released for commercialization in Brazil is a tetravalent, recombinant, chimeric live virus dengue vaccine called CYD-TDV that was developed by Sanofi Pasteur and marketed under the name Dengvaxia® [7]. Clinical trials involving CYD-TDV were conducted throughout the development stages of the vaccine in several countries and on different continents.

This work summarized the literature and estimated the efficacy of the commercially available dengue vaccine in Brazil to reduce symptomatic cases of dengue. Brazil has suffered an increasing burden of the disease, with 93.8% prevalence of serotype 1.

The question was addressed with: P- general population (adults and children), I- CYD-TDV (Dengvaxia®), C- placebo or other vaccines, O- symptomatic dengue, S- Clinical trials with comparators (Phase II-III).

## Methods

### Identification and selection of studies

A search for bibliographic references was performed through MEDLINE (via PubMed), LILACS (via Virtual Health Library), Cochrane Library (via Virtual Health Library) and EMBASE to locate randomized controlled trials that evaluated the efficacy of the dengue vaccine. The search was conducted between 2000 and 2017, which was the period corresponding to the most significant support for the development of vaccines against dengue with the tetravalent formulation of attenuated strains [8]. The search was limited to humans and performed without language restrictions.

The search strategies included the search for descriptors or words in the text related to the disease and the type of intervention. The complete search strategy is available in Additional file 1.

The inclusion considered phase II and phase III studies that evaluated the efficacy and safety of the tetravalent vaccine against dengue, studies that used a placebo or other vaccines as control, studies without gender or age restrictions and studies that reported the vaccine efficacy against clinically symptomatic dengue as an outcome. Studies with vaccines not commercially available in Brazil were excluded.

The evaluation of titles, abstracts, and the full text was independently performed by two reviewers (L.T.C.S. and I.C.); disagreements were examined and solved by a third reviewer (B.T.). The reviewers were blinded to the authors’ names when assessing the titles and abstracts. A manual search was performed on the references of the selected articles.

### Quality of evidence assessment

The bias risk assessment was independently performed by two reviewers (L.T.C.S. and B.T.) using the Cochrane Collaboration tool to assess the risk of bias from clinical trials [9]. The following criteria were evaluated: random sequence generation, allocation concealment, blinding of participants and personnel, blinding of outcome assessment, incomplete outcome data, and selective reporting. For other biases, we decided to evaluate the conflicts of interest.

### Data extraction

The outcomes of interest were primarily related to the efficacy of the dengue vaccine in preventing symptomatic dengue and, secondarily, those linked to specific prevention of each of the four serotypes. A standardized form was developed for data extraction with fields referring to the characteristics of identification of the studies, the countries involved, the study phase, the follow-up length, the patient ages, the observed outcomes and the number of person-years and the number of participants per intervention arm. Data regarding the seropositivity of the individuals were extracted at the beginning of the study.

### Statistical analysis of the data

The effect size of the intervention was estimated by the total of person-years, using the relative risk (RR) summary measure and the respective 95% confidence interval (95% CI). The efficacy was estimated to be [1-RR] and was expressed as a percentage. A meta-analysis was performed to estimate the overall efficacy and was stratified by higher impact variables. A meta-regression was performed to study the heterogeneity found in the meta-analysis.

The random-effects model was used with the results expressed as a percentage. A Forest plot-type chart was used to present the results of the meta-analysis and the comparison of the studies. The inconsistency (I^2^) method was used to assess heterogeneity among the studies.For the analyzes, the program R version 3.3.1 and the meta package version 4.4–1 were used.

## Results

**Fig. 1:**
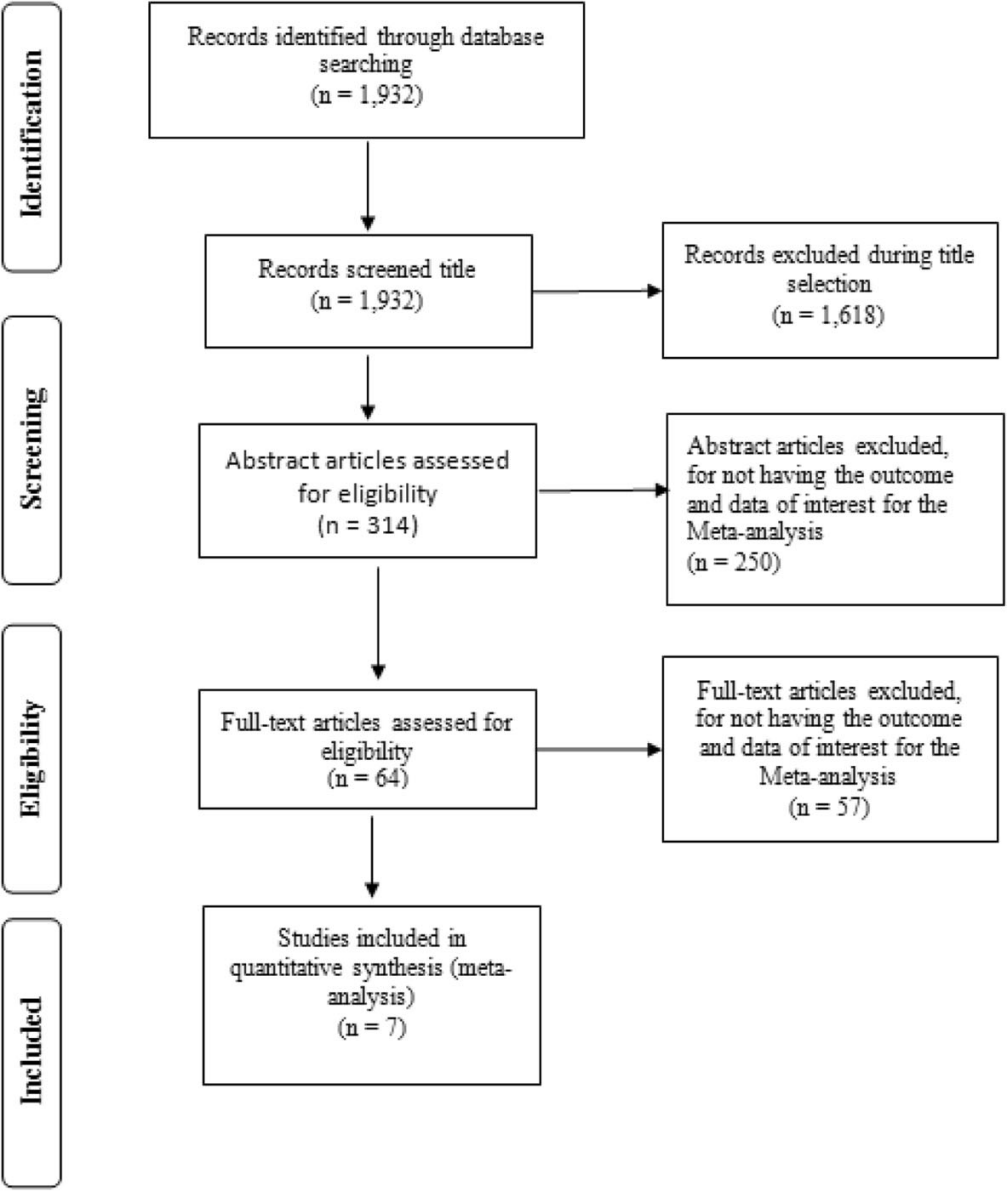
Summary of the selection phase results

A total of 1932 studies were identified in the surveyed databases, of which 1618 were eliminated by reading titles, 250 by reading the abstracts and 57 by reading the full text. Seven studies were selected for the analysis [10–16]. Figure 1 shows a summary of the selection phase results.

Of the 36,371 participants (66,511 person-years) in the selected studies, 30 were no longer randomized, 24,257 were randomized to the intervention group, and 12,144 were randomized to the control group. The population per trial ranged from 150 to 20,869, and the participants ranged in age from 2 to 45 years. Three studies were multinational [10, 11, 13] involving 4 to 5 countries in Latin America and Asia, and four studies involved only one country [12, 14–16], two of which were in Latin American countries (Brazil and Peru) [12, 15] and two of which were in Asia (Thailand and Vietnam) [14, 16]. Two studies included Brazilian patients and only children aged 9 to 16 years [10, 12], corresponding to 17% of the subjects in one study and 100% in the other study (Table 1).

**Table 1:** Characteristics of the included studies

Author	County	Design	Age (years)	Subjects	Follow-up time	Vaccinated	Dengue vaccine*	Control	Dengue Control**
Villar et al. [10]	Colombia, Brazil, Mexico, Honduras and Puerto Rico.	ECR phase III	9 to 16	20,869	25 months after the 1st dose	13,920	280	6949	388
Capeding et al. [11]	Indonesia, Malaysia, Philippines, Thailand and Vietnam.	ECR phase III	2 to 14	10,272	25 months after the 1st dose	6848	286	3424	309
Dayan et al. [12]	Brazil	ECR phase II	9 to 16	150	6 months after the 3rd dose	100	31	50	15
Villar et al. [13]	Colombia, Mexico, Honduras and Puerto Rico.	ECR phase II	9 to 16	600	6 months after the 3rd dose	401	43	199	29
Sabchareon et al. [16]	Thailand	ECR phase IIb	4 to 11	4002	25 months after the 1st dose	2669	76	1333	58
Tran et al. [14]	Vietnam	ECR phase II	2 to 45	180	6 months after the 3rd dose	120	4	60	3
Lanata et al. [15]	Peru	ECR phase II	2 to 11	298	1 month after the 3rd dose	199	1	99	3

*Cases of dengue in the vaccinated group

**Cases of dengue in the control group

The participants were followed-up for adverse events with mean follow-up times of 25 months in the phase III studies and 6 months in phase II studies. The phase III studies used placebo as a comparator, whereas the phase II studies used tetanus/diphtheria/acellular pertussis vaccines [13], the inactivated rabies vaccine [16], the anti-meningococcal A + C vaccine and the polysaccharide typhoid vaccine [14] and the polysaccharide pneumococcal vaccine [15]. All of the studies included the tetravalent, recombinant, live-attenuated dengue vaccine (CYD-TDV). All vaccine schedules included three doses administered at 0, 6 and 12 months. All the included studies were funded by the commercially available vaccine company.

**Fig. 2:**
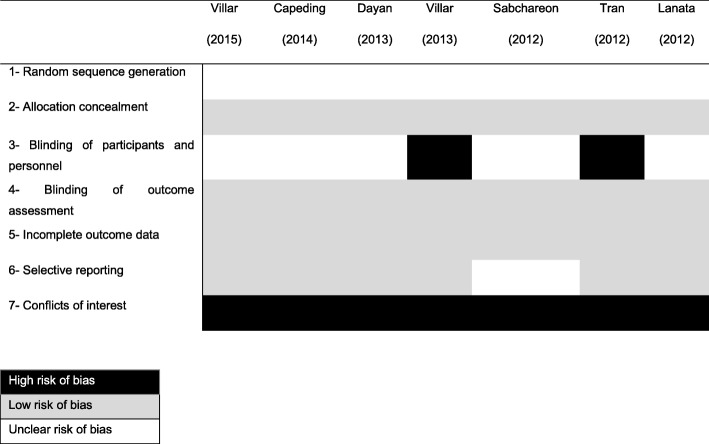
Risk of Bias

Regarding the bias risk analysis of the domains considered in the Cochrane Collaboration tool [9], all studies [10–16] presented a high bias risk for conflict of interest (sponsor performed the involved in critical steps as study design, sample testing, data analysis, data interpretation, and writing of the report). Two papers presented a high risk of bias for the masking of participants and professionals [13, 14]. In other domains, all the studies presented low risks of bias (Fig. 2).

Regarding the immunogenicity, most of the vaccinated individuals were previously immune to the disease as shown in Table 2, with seropositivity varying in the studies from 37 to 81%.

**Table 2:** Seropositivity at baseline

Study	Seropositivity of the vaccine group*	Seropositivity of the control group*
Dayan (2013)	69%	71%
Villar (2013)	75%	78%
Tran (2012)	71%	67%
Lanata (2012)	37%	48%
Sabchareon (2012)	70%	69%
Capeding (2014)**	68%	67%
Villar (2015)**	81%	77%

*Seropositivity of the participants at baseline

**Seropositivity was searched in the reactogenicity and immunogenicity subgroups, not in all participants

### Efficacy of the dengue vaccine

**Fig. 3:**
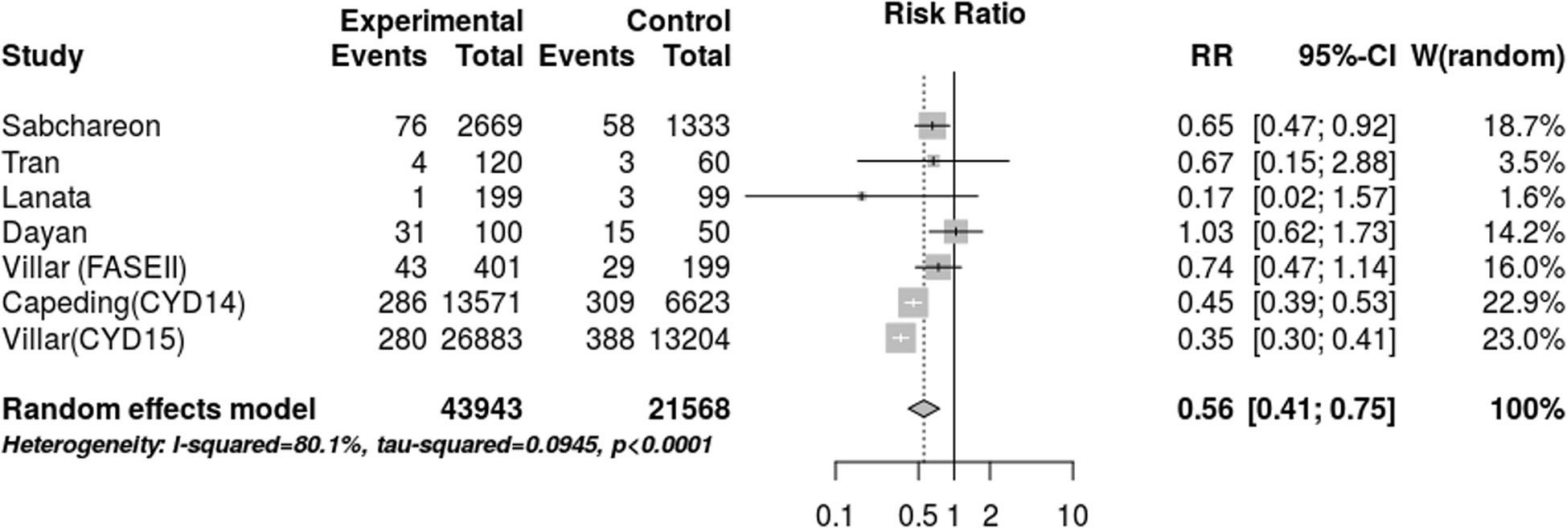
Meta-analysis of vaccine efficacy (cases person-years)

After the selection of articles and data collection, the meta-analysis was performed as shown in Fig. 3. In total, 36,371 participants (66,511 person-years) were included between the ages of 2 and 45 years. The random-effects model presented a RR of 0.56 (CI 0.41–0.75) with an I^2^ = 80.1% (*p* < 0.0001). The efficacy of the vaccine was estimated to be 44%, with a range from 25 to 59%. Two extensive studies [10, 11] dominated a considerable part of the outcome of the meta-analysis. A discrepancy was found between the results of these studies and the results of the studies with a smaller number of participants [12, 14]. Two of the clinical trials had wide confidence intervals [14, 15].

**Fig. 4:**
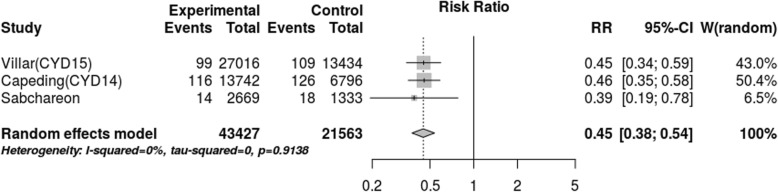
Efficacy for serotype 1 (cases persons-years)

**Fig. 5:**
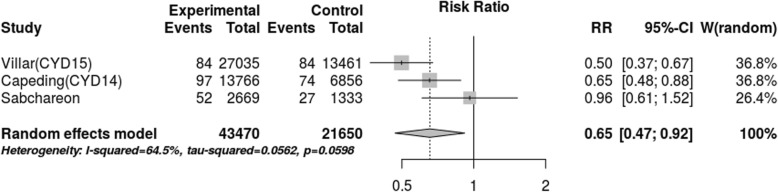
Efficacy for serotype 2 (cases persons-years)

**Fig. 6:**
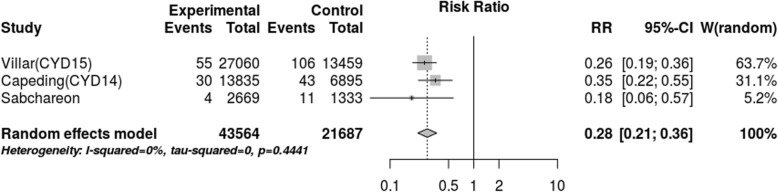
Efficacy for serotype 3 (cases persons-years)

**Fig. 7:**
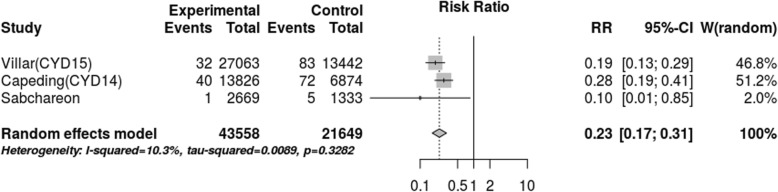
Efficacy for serotype 4 (cases persons-years)

After assessing the effect of seropositivity, virus type and age through meta-regression, we did not find an explanation for the 80.1% heterogeneity. However, the stratified meta-analysis (Figs. 4, 5, 6, 7), showed low heterogeneity (10.3%) for serotype 4 and significant heterogeneity (64.5%) for serotype 2. Therefore, the effect of the vaccine may not have been uniform by serotype, and this effect may have been responsible for the heterogeneity found in the analysis.

**Fig. 8:**
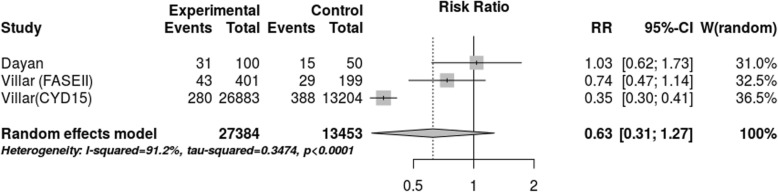
Efficacy in children (cases persons-years)

The meta-analysis using studies with efficacy data, above 9 years old, Fig. 8, the lower limit of age for the use of the vaccine, was the methodological option used for evaluating the effect of age in its efficacy. The estimated efficacy was even lower (37%), with a wide range of confidence of – 27 to 67%. Thus, besides efficacy not being statistically significant, the meta-analysis shows heterogeneity of 91%, indicating that it is still necessary to perform other studies for getting the measure of efficacy. It must be noted that the meta-analysis of Fig. 8 shows clear divergence between the estimation of efficacy of study CYD 15 and that of the other two studies, although being used the same dose of vaccine in the three studies, in such a way that the source of the heterogeneity of this meta-analysis is to be found in study CYD 15. More information about exclusions on Additional file 2 and the complete database on Additional file 2.

## Discussion

This systematic review allowed us to examine the evidence related to the primary efficacy outcome of the dengue vaccine against symptomatic dengue and the secondary efficacy outcome of the dengue vaccine against each of the viral serotype.

The data of phase II studies, from Dayan, Villar, Tran, and Lanata, and of phase IIb from Sabchareon, were included, being data of estimate of efficacy between treatment and control, with the allocation of therapies through randomization. Although these studies of phase II have been named as studies of safety and immunogenicity, they showed data of efficacy [17] and showed the minimum pre-requisites for being treated as clinical trials of phase III.

Regarding the efficacy of the vaccine against symptomatic dengue, the estimates indicated low protection when the whole set of studies was analysed, whereas the efficacy of the vaccine could not be proven in four studies when the studies were analysed separately.

Regarding the efficacy of the vaccine against each of the serotypes, three of the studies analysed the serotypes separately in the intention-to-treat analysis and obtained similar results. These studies showed high efficacy against serotypes 3 and 4 and much lower efficacy for serotypes 1 and 2, with the efficacy for serotype 2 being markedly lower than the efficacy against the other strains. Overall estimates of vaccine efficacy show a tendency for modest protection, whereas the results by serotype (related to serotypes 1 and 2) do not show vaccine protection because they are not significant.

Since randomization was only warranted in the intention-to-treat analysis, the per-protocol analyses, which could be biased, were not considered. Because the primary goal of the vaccine in the studies was effectiveness against symptomatic dengue and virological confirmation, many cases of asymptomatic infection were not detected, which further reduced the vaccine efficacy.

When considering the efficacy for the serotypes, there was a predominance of viral serotypes 1 and 4 (93.8 and 5.1%, respectively) compared to serotypes 2 and 3 (0.7 and 0.4%, respectively) in Brazil in 2015 [18]. Thus, the vaccine demonstrated non-significant results for serotype 1, which was the serotype with the largest circulation in Brazil in 2015. This phenomenon could be related to a lack of efficacy for serotype 2.

Studies in Asia have shown greater efficacy of the vaccine for children 9 years of age and low efficacy for children between 2 and 5 years of age, which can be explained by the greater seropositivity as children grow and acquire active immunity against the disease through living in endemic areas and areas with high viral circulation. In studies in Latin America, this observation could not be made because the studies included age groups 9 years or older; the results of these studies were more efficacious among those seropositive at the beginning of the studies. Since vaccination is a preventive strategy, ideally the vaccine should be effective for age groups of less than 9 years and be independent of the previous seropositivity.

Another relevant finding for the vaccine is the proposed vaccination schedule of three doses with 6-month intervals between doses. This dosing schedule can lead to incomplete vaccinations, which is inevitable in a schedule of repeated doses with a considerable time interval between them in a population that in some areas has limited access to health care. The effect could be much lower efficacy than the already reduced efficacy. Some vaccines, such as the HPV vaccine, have a 55% loss of adherence [19].

The reduction in hospitalization rates and dengue hemorrhagic fever in the phase III study in Asia and Latin America should be viewed with caution. The criteria for hospitalization differ between countries, and these criteria may vary by location even within the same country. Because no clear definition was available concerning what constituted hospitalization in the studies, hospitalization was considered a hospital stay excluding short-term emergency care. In Brazil, many municipalities rely on family health coverage and emergency care units as a back-up. These units are responsible for the care of the majority of dengue cases, including venous hydration in hydration armchairs, without hospitalization. Thus, the hospitalization data for dengue are not counted. These care arrangements may make it difficult to measure whether hospitalization is reduced due to the vaccine or the care structure.

Another important factor regarding reducing the rates of hospitalization and dengue hemorrhagic fever is that the seroprevalence was not known at the beginning of the studies. Because the seroprevalence was only noted in one study [16], this factor could have interfered with the findings.

In Brazil, two new arboviruses (Chikungunya and Zika) are circulating, which makes vector control a permanent task regarding logistics and cost.

The dengue vaccine has recently been commercialized, but no sufficient studies with follow-up times are available to fill the existing gaps in the studies included in this review. We expect that new studies will be published that can be added to the meta-analysis performed here.

Questions related to long-term safety and efficacy must be answered, especially those concerning the possibility of a more severe clinical picture of dengue, particularly in vaccinated individuals who do not have an immune response to DENV-2 but who produce antibodies against this serotype. Significant adverse events could be triggered by the vaccine, especially severe dengue, once the vaccine acts on one serotype when it is desirable a global efficacy for all serotypes.

Consideration should also be given to the post-marketing period and all events inherent to this time frame, especially those linked to safety and pharmacovigilance.

The efficacy of 44% may be considered low, especially when compared to the efficacy of approximately 95% [17] reported for the vaccines against yellow fever, hepatitis B, rubella, measles and mumps and the efficacy of 100% for the tetanus vaccine.

The efficacy of the dengue vaccine was also the object of two other systematic reviews in Brazil [20, 21], and a study of efficacy using pooled data [22].

The study by Costa et al. [20] included only phase II studies for the efficacy meta-analysis. Godói et al. [21] included 9 studies, 6 phase II studies and 3 phase III studies but used only the 2 major phase III studies. Hadinegoro et al. [22] included 1 phase II study and 2 phase III studies. Thus, the three studies obtained results different from those of the present study, which included and used the 7 phase II and III studies. The authors from our study found no reason to exclude the 5 phase II articles [12–16], that used the same treatment and dose of dengue vaccine CYD-TDV.

## Conclusions

The results of the meta-analysis presented in this review showed low efficacy of the vaccine against symptomatic dengue, especially against serotypes 1 and 2. We anticipate a limited impact of the use of the CYD-TDV vaccine as a primary prevention strategy for the disease.

## Additional files

Additional file 1: Database search strategy. Includes a detailed description of the search strategy for Medline, Cochrane and Lilacs (DOC 35 kb)

Additional file 2: Exclusion list. A list of the reason for all exclusions (DOC 138 kb)

Additional file 3: Dataset_dengue. The complete systematic review dataset (CSV 1 kb)

## Data Availability

The dataset supporting the conclusions of this article is the Additional file 3.

## Abbreviations

CI: confidence interval
CYD-TDV: a tetravalent, recombinant, chimeric live virus dengue vaccine
DENV-1, DENV-2, DENV-3, and DENV-4: serotypes of Dengue viruses
RR-: relative risk
WHO: World Health Organization
