# Supplementary material for: Systematic review of dengue vaccine efficacy
Source: BMC Infect Dis. 2019 Aug 28;19:750. doi: 10.1186/s12879-019-4369-5 (PMC6712597; doi:10.1186/s12879-019-4369-5)
Supplement: Supplementary file 1 — Database search strategy. Includes a detailed description of the search strategy for Medline, Cochrane and Lilacs (DOC 35 kb) [file 12879_2019_4369_MOESM1_ESM.doc]

**Additional file 1.** Database search strategy.

| Data base | Search strategy |
| --- | --- |
| Medline (PUBMED) | (((vaccines[Mesh] or vaccin*[tw] or immunization[Mesh] or immuniz*[tw] or immunis*[tw]) and (Dengue[Mesh] or Dengue[tw])) or "Dengue Vaccine"[Mesh]) not (animals[Mesh] not humans[Mesh]) |
| COCHRANE | Dengue |
| LILACS | (vacina da dengue) or (vacina contra a dengue) or (dengue vaccines) or (vacunas contra el dengue) or (D.20.215.894.899.162) AND NOT (animals) |
| EMBASE | (('vaccine'/exp or 'vaccine' or vaccine:ab, ti or 'immunization'/exp or immunization or immunization:ab, ti and ('dengue virus'/exp or 'dengue virus' or dengue:ab, ti) or ('dengue'/exp or dengue and ('vaccine'/exp or vaccine))) and [embase]/lim not [medline]/lim and ([cochrane review]/lim or [systematic review]/lim or [meta-analysis]/lim or [controlled clinical trial]/lim or [randomized controlled trial]/lim)) and ('article'/it or 'article in press'/it or 'review'/it) |
