# Supplementary material for: Systematic review of dengue vaccine efficacy
Source: BMC Infect Dis. 2019 Aug 28;19:750. doi: 10.1186/s12879-019-4369-5 (PMC6712597; doi:10.1186/s12879-019-4369-5)
Supplement: Supplementary file 2 — Exclusion list. A list of the reason for all exclusions (DOC 138 kb) [file 12879_2019_4369_MOESM2_ESM.doc]

**Additional file 2 Exclusion list**

| Author (year) | Title | Place of publication | **Reason for exclusion** |
| --- | --- | --- | --- |
| Durbin 2016 | A 12-month interval dosing study in adults indicates that a single dose of NIAID tetravalent dengue vaccine induces a robust neutralizing antibody response | J.Infect.Dis | Absence of the selected clinical outcome |
| Gessner 2016 | Estimating the public health importance of the CYD-tetravalent dengue vaccine: vaccine preventable disease incidence and numbers needed to vaccinate | Vaccine | Absence of the selected clinical outcome |
| Hernández-Ávila 2016 | Análisis de la evidencia sobre eficacia y seguridad de la vacuna de dengue CYD-TDV y su potencial registro e implementación em el programa de vacunación universal de México | Salud.Públ.Méx. | Absence of the selected clinical outcome |
| Plennevaux 2016 | Detection of dengue cases by serological testing in a dengue vaccine efficacy trial: Utility for efficacy evaluation and impact of future vaccine introduction | Vaccine | Absence of the selected clinical outcome |
| Sirivichayakul 2016 | Safety and Immunogenicity of a Tetravalent Dengue Vaccine Candidate in Healthy Children and Adults in Dengue-Endemic Regions: A Randomized, Placebo-Controlled Phase 2 Study | J Infect Dis | Absence of the selected clinical outcome |
| Vannice 2016 | Status of vaccine research and development of vaccines for dengue. | Vaccine | Absence of the selected clinical outcome |
| Watanaveeradej 2016 | Long-term safety and immunogenicity of a tetravalent live-attenuated dengue vaccine and evaluation of a booster dose administered to healthy thai children | Am. J. Trop. Med | Technology different from that analyzed |
| Wilder-Smith 2016 | Age specific differences in efficacy and safety for the CYD-tetravalent dengue vaccine | Expert Rev Vaccines | Absence of the selected clinical outcome |
| Chu 2015 | CD8+ T-cell Responses in Flavivirus-Naive Individuals Following Immunization with a Live-Attenuated Tetravalent Dengue Vaccine Candidate. | J.Infect.Dis | Absence of the selected clinical outcome |
| Coudeville 2015 | Estimation of parameters related to vaccine efficacy and dengue transmission from two large phase III studies | Vaccine | Absence of the selected clinical outcome |
| Hadinegoro 2015 | Efficacy and Long-Term Safety of a Dengue Vaccine in Regions of Endemic Disease | N Engl J Med | Absence of primary data. |
| Jelitha 2015 | Descriptive Review of Safety, Reactogenicity and Immunogenicity of Dengue Vaccine Clinical Trials, 2003 - 2013. | Med J Malaysia | Absence of the selected clinical outcome |
| Kirkpatrick 2015 | Robust and balanced immune responses to all 4 dengue virus serotypes following administration of a single dose of a live attenuated tetravalent dengue vaccine to healthy, flavivirus-naive adults | J.Infect.Dis | Absence of the selected clinical outcome |
| Lee 2015 | A multi-country study of the household willingness-to-pay for dengue vaccines: household surveys in Vietnam, Thailand, and Colombia | ‎PLOS Negl. Trop. Dis | Absence of the selected clinical outcome |
| Pandey 2015 | The introduction of dengue vaccine may temporarily cause large spikes in prevalence. | Epidemiol Infect | Absence of the selected clinical outcome |
| Precioso 2015 | Clinical evaluation strategies for a live attenuated tetravalent dengue vaccine. | Vaccine | Technology different from that analyzed |
| Torresi 2015 | Lot-to-lot consistency of a tetravalent dengue vaccine in healthy adults in Australia: a randomised study. | Vaccine | Absence of the selected clinical outcome |
| Tsai 2015 | Complexity of Neutralizing Antibodies against Multiple Dengue Virus Serotypes after Heterotypic Immunization and Secondary Infection Revealed by In-Depth Analysis of Cross-Reactive Antibodies. | J. Virol. | Absence of the selected clinical outcome |
| Weiskopf 2015 | The human CD8+ T cell responses induced by a live attenuated tetravalent dengue vaccine are directed against highly conserved epitopes. | J. Virol. | Absence of the selected clinical outcome |
| Costa 2014 | Safety, immunogenicity and efficacy of a recombinant tetravalent dengue vaccine: a meta-analysis of randomized trials. | Vaccine | Absence of primary data. |
| Jindal 2014 | Dengue vaccine: a valuable asset for the future. | Hum Vaccin Immunother | Absence of the selected clinical outcome |
| Mbah 2014 | Country- and age-specific optimal allocation of dengue vaccines. | J. Theor Biol | Absence of the selected clinical outcome |
| NdeffoMbah 2014 | Country- and age-specific optimal allocation of dengue vaccines | J Theor Biol | Absence of the selected clinical outcome |
| Thavara 2014 | Simulations to compare efficacies of tetravalent dengue vaccines and mosquito vector control. | Epidemiol Infect | Absence of the selected clinical outcome |
| Baernighausen 2013 | Valuing the broader benefits of dengue vaccination, with a preliminary application to Brazil | Semin Immunol | Absence of the selected clinical outcome |
| Durbin 2013 | A single dose of any of four different live attenuated tetravalent dengue vaccines is safe and immunogenic in flavivirus-naive adults: a randomized, double-blind clinical trial. | J Infect Dis | Absence of the selected clinical outcome |
| Durham 2013 | Dengue dynamics and vaccine cost-effectiveness in Brazil. | Vaccine | Absence of the selected clinical outcome |
| Haremberg 2013 | Persistence of Th1/Tc1 responses one year after tetravalent dengue vaccination in adults and adolescents in Singapore. | Hum Vaccin Immunother | Absence of the selected clinical outcome |
| HSS 2013 | Safety and immunogenicity of a tetravalent dengue vaccine in healthy children aged 2-11 years in Malaysia: a randomized, placebo-controlled, Phase III study. | Vaccine | Absence of the selected clinical outcome |
| Ramachanderam 2013 | Descriptive review of safety, reactogenicity and imunogenicity of dengue vaccine clinical trials, 2003 - 2013. | Med. J. Malaysia | Absence of the selected clinical outcome |
| Sun 2013 | Experimental dengue virus challenge of human subjects previously vaccinated with live attenuated tetravalent dengue vaccines. | J Infect Dis | Absence of the selected clinical outcome |
| Chao 2012 | Controlling dengue with vaccines in Thailand | ‎PLOS Negl Trop Dis | Absence of the selected clinical outcome |
| Coudeville 2012 | Transmission dynamics of the four dengue serotypes in Southern Vietnam and potencial impact of vaccination | PLoS One | Absense of the selected clinical outcome |
| Leo 2012 | Immunogenicity and safety of recombinant tetravalent dengue vaccine (CYD-TDV) in individuals aged 2-45 years. Phase II rabdomized controlled trial in Singapore | Hum Vaccin Immunother | Absence of the selected clinical outcome |
| Mahoney 2012 | Cost of production of live attenuated dengue vaccines: a case study of the Instituto Butantan, Sao Paulo, Brazil. | Vaccine | Technology different from that analyzed |
| Amarasinghe 2011 | Estimating potential demand and supply of dengue vaccine in Brazil. | Hum Vaccin | Absence of the selected clinical outcome |
| Carrasco 2011 | Economic impact of dengue illness and the cost-effectiveness of future vaccination programs in Singapore. | ‎PLOS Negl. Trop. Dis | Absence of the selected clinical outcome |
| Durbin 2011 | Heterotypic dengue infection with live attenuated monotypic dengue virus vaccines: implications for vaccination of populations in areas where dengue is endemic. | J Infect Dis | Absence of the selected clinical outcome |
| Guy 2011 | From research to phase III: preclinical, industrial and clinical development of the Sanofi Pasteur tetravalent dengue vaccine. | Vaccine | Absence of the selected clinical outcome |
| Lee 2011 | Economic value of dengue vaccine in Thailand. | Am J Trop Med Hyg | Absence of the selected clinical outcome |
| Murrell 2011 | Review of dengue virus and the development of a vaccine | Biotechnol Adv | Absence of the selected clinical outcome |
| Amarasinghe 2010 | Forecasting dengue vaccine demand in disease endemic and non-endemic countries. | Hum Vaccin | Absence of the selected clinical outcome |
| Guy 2010 | Development of Sanofi Pasteur tetravalent dengue vaccine. | Hum Vaccin | Absence of the selected clinical outcome |
| Morrison 2010 | A novel tetravalent dengue vaccine is well tolerated and immunogenic against all 4 serotypes in flavivirus-naive adults | J Clin Virol | Absence of the selected clinical outcome |
| Trent 2010 | WHO Working Group on technical specifications for manufacture and evaluation of dengue vaccines, Geneva, Switzerland, 11-12 May 2009 | Vaccine | Absence of the selected clinical outcome |
| Guy 2009 | Immunogenicity of sanofi pasteur tetravalent dengue vaccine | J Clin Virol | Absence of the selected clinical outcome |
| Edelman 2008 | Guidelines for the clinical evaluation of dengue vaccines in endemic areas": summary of a World Health Organization Technical Consultation | Vaccine | Absence of the selected clinical outcome |
| Palanca-Tan 2008 | The demand for a dengue vaccine: a contingent valuation survey in Metro Manila. | J Infect Dis | Absence of the selected clinical outcome |
| Simasathien 2008 | Safety and immunogenicity of a tetravalent live-attenuated dengue vaccine in flavivirus naive children | Am J Trop Med Hyg | Absence of the selected clinical outcome |
| Chanthavanich 2006 | Short report: immune response and occurrence of dengue infection in Thai children three to eight years after vaccination with live attenuated tetravalent dengue vaccine | Am J Trop Med Hyg | Absence of the selected clinical outcome |
| Guy 2004 | Evaluation by flow cytometry of antibody-dependent enhancement (ADE) of dengue infection by sera from Thai children immunized with a live-attenuated tetravalent dengue vaccine. | Vaccine | Absence of the selected clinical outcome |
| Sabchareon 2004 | Safety and immunogenicity of a three dose regimen of two tetravalent live-attenuated dengue vaccines in five- to twelve-year-old Thai children | Pediatr Infect Dis J | Absence of the selected clinical outcome |
| Shepard 2004 | Cost-effectiveness of a pediatric dengue vaccine. | Vaccine | Absence of the selected clinical outcome |
| Sun 2003 | Vaccination of human volunteers with monovalent and tetravalent live-attenuated dengue vaccine candidates | Am J Trop Med Hyg | Absence of the selected clinical outcome |
| Sabchareon 2002 | Safety and immunogenicity of tetravalent live-attenuated dengue vaccines in Thai adult volunteers: role of serotype concentration, ratio, and multiple doses | Am J Trop Med Hyg | Absence of the selected clinical outcome |
| Kanesa-thasan2001 | Safety and immunogenicity of attenuated dengue virus vaccines (Aventis Pasteur) in human volunteers. | Vaccine | Absence of the selected clinical outcome |
| Hoke 1990 | Preparation of an attenuated dengue 4 (341750 Carib) virus vaccine. II. Safety and immunogenicity in humans. | Am J Trop Med Hyg | Absence of the selected clinical outcome |
